# Characterization of Hydrogen Metabolism in the Multicellular Green Alga *Volvox carteri*

**DOI:** 10.1371/journal.pone.0125324

**Published:** 2015-04-30

**Authors:** Adam J. Cornish, Robin Green, Katrin Gärtner, Saundra Mason, Eric L. Hegg

**Affiliations:** 1 Great Lakes Bioenergy Research Center and the Department of Biochemistry & Molecular Biology, Michigan State University, East Lansing, Michigan, United States of America; Donald Danforth Plant Science Center, UNITED STATES

## Abstract

Hydrogen gas functions as a key component in the metabolism of a wide variety of microorganisms, often acting as either a fermentative end-product or an energy source. The number of organisms reported to utilize hydrogen continues to grow, contributing to and expanding our knowledge of biological hydrogen processes. Here we demonstrate that *Volvox carteri* f. *nagariensis*, a multicellular green alga with differentiated cells, evolves H_2_ both when supplied with an abiotic electron donor and under physiological conditions. The genome of *Volvox carteri* contains two genes encoding putative [FeFe]-hydrogenases (*HYDA1* and *HYDA2*), and the transcripts for these genes accumulate under anaerobic conditions. The *HYDA1* and *HYDA2* gene products were cloned, expressed, and purified, and both are functional [FeFe]-hydrogenases. Additionally, within the genome the *HYDA1* and *HYDA2* genes cluster with two putative genes which encode hydrogenase maturation proteins. This gene cluster resembles operon-like structures found within bacterial genomes and may provide further insight into evolutionary relationships between bacterial and algal [FeFe]-hydrogenase genes.

## Introduction

Hydrogen is an essential component in the metabolism of a variety of microorganisms [1,2,3]. In biology, the production of H_2_ is predominantly catalyzed by two classes of enzymes, hydrogenases and nitrogenases [1,4], with hydrogenases also contributing to H_2_ uptake [2]. Microbes are able to use these enzymes to catalyze the oxidation of H_2_ and/or the reduction of protons during fermentation [2,5,6,7]. In addition, recent reports suggest that H_2_ may also play a role in deacidification, enhancing the cell viability of certain microbes in harsh environments [8,9,10]. The complex role of H_2_ in the life-cycle of autotrophic microorganisms, such as green algae, is linked to both photosynthetic and fermentative processes [11,12], and studies of H_2_ metabolism in these microbes establish a basis for future metabolic and evolutionary studies of algal species which are currently uncharacterized in biohydrogen production [12,13,14,15,16].

Green algae both evolve and consume H_2_ using [FeFe]-hydrogenases (EC#1.12.7.2), metalloproteins capable of catalyzing the reduction of protons as well as the oxidation of H_2_ [5,6,17,18]. Electrons for H_2_ production can either be channeled from photosynthetic water-splitting or obtained by the fermentation of carbon sources [6,19]. In a variety of green algae, hydrogenases are encoded by two gene paralogs (*HYDA1* and *HYDA2*) that have high sequence similarity. Although the respective physiological functions of each enzyme are still unclear [20,21], there is evidence suggesting that the *HYDA1* gene product may contribute more to H_2_ production in the light [22]. Maturation proteins are required to assemble the catalytic active site, and the corresponding genes (*HydE*, *HydF*, and *HydG* in bacteria—*HYDEF* and *HYDG* in green algae) are ubiquitous in the genomes of organisms which utilize [FeFe]-hydrogenase [23]. Although ancestral forms of these genes in green algae were likely acquired by lateral gene transfer during evolution [24,25,26,27], hydrogenase gene clustering has not been noted in sequenced green algal genomes. Despite this lack of clustering, hydrogenase and maturation factor gene expression is tightly co-regulated. In addition, [FeFe]-hydrogenases are irreversibly-inactivated by the presence of O_2_, and expression of the *HYD* genes is induced under anaerobiosis [5,14].

The O_2_-sensitivity of [FeFe]-hydrogenases renders H_2_ synthesis dependent on micro-aerobic conditions and thus limits production during light-driven oxygenic photosynthesis [15,28,29]. In *Chlamydomonas reinhardtii*, a model unicellular green alga, this inhibition may be overcome by sulfur deprivation, which limits O_2_ production by photosystem II while still allowing electrons gained from photosystem I to be coupled to H_2_ production via chloroplastidic ferredoxins [13]. In addition, anaerobic conditions are quickly established in the dark as respiration depletes O_2_, thereby allowing carbon stores generated during photosynthesis to be utilized for H_2_ production [15,30]. Both of these methods allow for channeling of electrons to H_2_ synthesis.

*Volvox carteri* is a multicellular green alga that is separated from *C*. *reinhardtii* by approximately 220 million years of evolution [31]. *V*. *carteri* is composed of two cell types, gonidia and somatic, which are embedded within an extracellular matrix [32,33]. Consistent with the high degree of sequence similarity between the genomes of *V*. *carteri* and *C*. *reinhardtii* [31], these two organisms appear to share a number of similar metabolic processes [34]. *V*. *carteri* has been studied for >40 years [35] and H_2_ production has previously been observed from a *Volvox* species [36]. Recently, two putative [FeFe]-hydrogenase genes were annotated in the genome of *V*. *carteri* [31], which may provide the organism with H_2_ metabolism similar to that of *C*. *reinhardtii*.

In this manuscript, we provide the first report of both *in vitro* and *in vivo* H_2_ production in the multicellular green alga, *V*. *carteri* f. *nagariensis* and demonstrate that functional [FeFe]-hydrogenases are encoded by the annotated *HYDA* genes. Genes coding for the functional hydrogenases and associated maturation factors are arranged in a unique operon-like gene cluster within the *V*. *carteri* genome, providing additional evidence for an evolutionary relationship between bacterial and green algal [FeFe]-hydrogenases. Together, these data support a role for H_2_ in the metabolism of *V*. *carteri* and provide a basis for further investigation of the ancestral acquisition of [FeFe]-hydrogenase genes in green algae.

## Experimental

### Growth Conditions

Unless otherwise noted, *V*. *carteri* f. *nagariensis* EVE [31] cells were grown autotrophically under continuous light (90 μE m^-2^ s^-1^) and shaking (100 rpm) without bubbling. All cultures were incubated at 22°C in standard *Volvox* medium (SVM), a minimal medium modified from Kirk et al. [37] and Yamano et al. [38] and primarily composed of metal salts [0.5 mM Ca(NO_3_)_2_ 4H_2_O, 0.16 mM MgSO_4_ 7H_2_O, 0.16 mM sodium glycerophosphate pentahydrate, 0.67 mM KCl, 3.8 mM glycylglycine, 99.6 nM vitamin B_12_ (dissolved in 50 mM HEPES (pH 7.8)), 1.02 μM biotin (dissolved in 50 mM HEPES (pH 7.8)), and 6 mL of P-IV Metal Solution (2 mM Na_2_EDTA 2H_2_O, 0.36 mM FeCl_3_ 6H_2_O, 0.21 mM MnCl2 4H_2_O, 0.037 mM ZnCl_2_, 0.0084 mM CoCl_2_ 6H_2_O, 0.018 mM Na_2_MoO_4_ H_2_O) per liter of media].

For physiological H_2_ production assays, *V*. *carteri* cultures were incubated with 20 mM sodium bicarbonate for 72 hours prior to anaerobic acclimation to increase CO_2_ levels for improved carbon fixation.

### Anaerobic Acclimation

To acclimate *V*. *carteri* cells to anaerobiosis, a culture of light-adapted cells was centrifuged at 1,000× *g* for 5 min. The resulting cell pellet was washed three times in an anaerobic chamber (Coy Laboratory Products) with anaerobic media (degassed SVM supplemented with 0.16 mM MgCl_2_ rather than 0.16 mM MgSO_4_ to exclude sulfate from the medium). Cell shading is often sufficient to significantly reduce oxygenic photosynthesis in thick cultures of unicellular green alga. However, due to non-homogenous mixing of *V*. *carteri* cultures and the low densities of individual cells within spheroids, the vials were wrapped in aluminum foil to establish dark conditions. To prevent the possibility of residual oxygenic photosynthetic activity from stray light, sulfate-depleted medium was also employed to decrease photosystem II activity, a strategy successfully utilized previously in experiments with *C*. *reinhardtii* [39,40,41]. The cells were resuspended in degassed media to a final concentration of 75 μg chlorophyll/mL, and 2 mL of this mixture were sealed in a 10 mL air-tight serum vial (Wheaton). The resuspension was shaken for 4 h before further experimentation.

### Hydrogen Production Measurements

H_2_ evolution using an artificial electron donor was measured by incubating 0.1 mL of either aerobically- or anaerobically-acclimated cells (75 μg chlorophyll/mL) with 1.9 mL of H_2_ evolution assay solution [degassed media, 100 mM sodium dithionite, 10 mM methyl viologen dichloride (MV)] in a sealed 10 mL serum vial at 22°C in the dark with continuous shaking. At various time points, 20 μL of headspace gas were injected into a TRACE GC Ultra Gas Chromatograph (Thermo Scientific) using a 100 μL syringe. The peak at 1.4 min corresponded to H_2_, and the absolute value of H_2_ gas was determined by comparison to a standard curve. The resulting values were plotted versus time to monitor the accumulation of H_2_ in the headspace, and the rates were determined per μg of chlorophyll.

Selected previous reports on H_2_ production in algae have utilized detergents to rupture cells and allow the hydrogenase proteins access to the abiotic electron donors. Initial experiments demonstrated no significant difference upon addition of detergent (data not shown), suggesting that detergent is not essential under our assay conditions.

### Cell Separation

All steps for the cell separation were performed anaerobically using a protocol adapted from Nematollahi et al. [42]. Briefly, an anaerobically-acclimated 1 L culture was centrifuged at 1,800× g in air-tight 250 mL centrifuge bottles (Nalgene) for 10 min. The supernatant was discarded and the pellet was resuspended in 30 mL of anaerobic media. The suspension was passed through a 100 μm filter (Genesee Scientific) and the flow-through discarded. The cells were washed off of the filter in 10 mL of anaerobic media and the cells were liberated from the extracellular matrix using a 50 mL glass Dounce homogenizer (Sigma-Aldrich), moving the pestle up and down 10 times. An additional 20 mL of anaerobic media was added to the suspension and the gonidia were allowed to settle for 10 min. The top 20 mL of the suspension enriched in somatic cells was passed successively through 30 μm and 10 μm meshes (Genesee Scientific and Membrane Solutions, respectively). Following passage through the 10 μm mesh, the flow-through contained pure somatic cells as determined by microscopy.

The settled cells enriched in gonidia were resuspended in 10 mL of anaerobic media and passed through a 100 μm mesh. The flow-through was then passed through a 30 μm mesh, collecting the cells on the filter. The purified gonidia were washed off of the filter and resuspended in 2 mL of anaerobic media. The homogeneity of the gonidia samples was confirmed by microscopy.

### RNA Isolation

To isolate total RNA from *V*. *carteri*, cell cultures were centrifuged at 10,000× *g* for 10 min. The resulting pellets were resuspended in 0.5 mL of TRIzol (Invitrogen) and frozen in liquid nitrogen. The frozen TRIzol/cell mass mixture was crushed using a mortar and pestle, thawed, and resuspended with an additional 0.5 mL of TRIzol. To this suspension, 0.2 mL of chloroform was added, and the mixture was vortexed at top speed at room temperature for 15 min. To pellet cell debris, the suspension was centrifuged at 10,000× *g* for 15 min at 10°C. The aqueous phase was transferred to a 1.7 mL tube (Denville) containing 0.25 mL of 0.8 M sodium citrate and 1.0 M sodium chloride, and the suspension was gently mixed by inversion. Following the addition of 0.25 mL of isopropanol, the mixture was centrifuged at 10,000× *g* for 25 min at 10°C. The supernatant was discarded and the RNA pellet washed twice with ice-cold 75% ethanol. The pellet was allowed to dry and was then resuspended in 20 μL of diethylpyrocarbonate-treated water.

### RT-PCR

Reverse transcription of mRNA was performed using M-MLV Reverse Transcriptase (Invitrogen), and the cDNA was amplified for 30 cycles via PCR using GoTaq Green Master Mix (Promega) and the primer sets listed in Table 1. Genomic and cDNA sequences of *HYDA1*, *HYDA2*, *HYDEF*, and *HYDG* can be accessed from the DDBJ/EMBL/GenBank database with the accession numbers XM_002948441, XM_002948437, XM_002948568, and XM_002948439, respectively.

**Table 1:** List of primers used for cloning and cDNA amplification. Restriction digest sites are *italicized* and 6xHis-tag sequences are underlined. Genomic sequences amplified by the cloning primers are in bold.

**Cloning**	**Sequence**
*HYDA1* Sense	GCGCGC *CCATGG* CG CAT CAC CAT CAC CAT CAC GGTGGCGGA **ATGGACGAGCTAGACAAGCC**
*HYDA1* Antisense	GCGCGC AAGCTT **TCTACTCGGCCTCGACACCA**
*HYDA2* Sense	GCGCGC *CCATGG* GA CAT CAC CAT CAC CAT CAC GGAGGCGGT **AAGTGCACTTCGGCTGTCC**
*HYDA2* Antisense	GCGCGC *AAGCTT* **ACTCGGTATCGACGCCC**
**RT-PCR**	**Sequence**
*HYDA1* Sense	ATGGACGAGCTAGACAAGCC
*HYDA1* Antisense	TCTACTCGGCCTCGACACCA
*HYDA2* Sense	TAAGTGCACTTCGGCTGTCC
*HYDA2* Antisense	TTACTCGGTATCGACGCC
*ACTA* Sense	ATGGCTGAGGAGGGCGAGGT
*ACTA* Antisense	TTAGAAGCACTTCCGGTGCA

### Cloning of *HYDA1* and *HYDA2*

The *HYDA1* and *HYDA2* genes were PCR amplified with *PfuTurbo* DNA polymerase (Stratagene) from reverse-transcribed cDNA using the primers listed in Table 1. The amplified products were ligated into the SacI/HindIII site of pAC-BAD, a pBAD/D-TOPO (Invitrogen) expression vector (contains a kanamycin-resistant cassette and an l-arabinose inducible promoter) that was modified to remove the N-terminal thioredoxin-tag [43]. To achieve optimal yields of active protein (using a heterologous expression system previously described [43,44]), the constructs were transformed into *S*. *oneidensis* MR-1 Δ*hydA/*Δ*hyaB* [45] electrocompetent cells as detailed by Ozawa [46] and selected for antibiotic resistance on 50 μg/mL kanamycin sulfate LB plates.

### Overexpression, Protein Purification, and Hydrogen Evolution Assay

Cells harboring the pAC-BAD_*HYDA1* and pAC-BAD_*HYDA2* vectors were induced for gene overexpression and protein synthesis as previously described [43]. Enzymes were purified from *S*. *oneidensis* cultures in an anaerobic chamber and assayed for H_2_ evolution activity as described by Cornish et al. [43].

## Results

### In vivo and in vitro H_2_ Evolution

**Fig 1:**
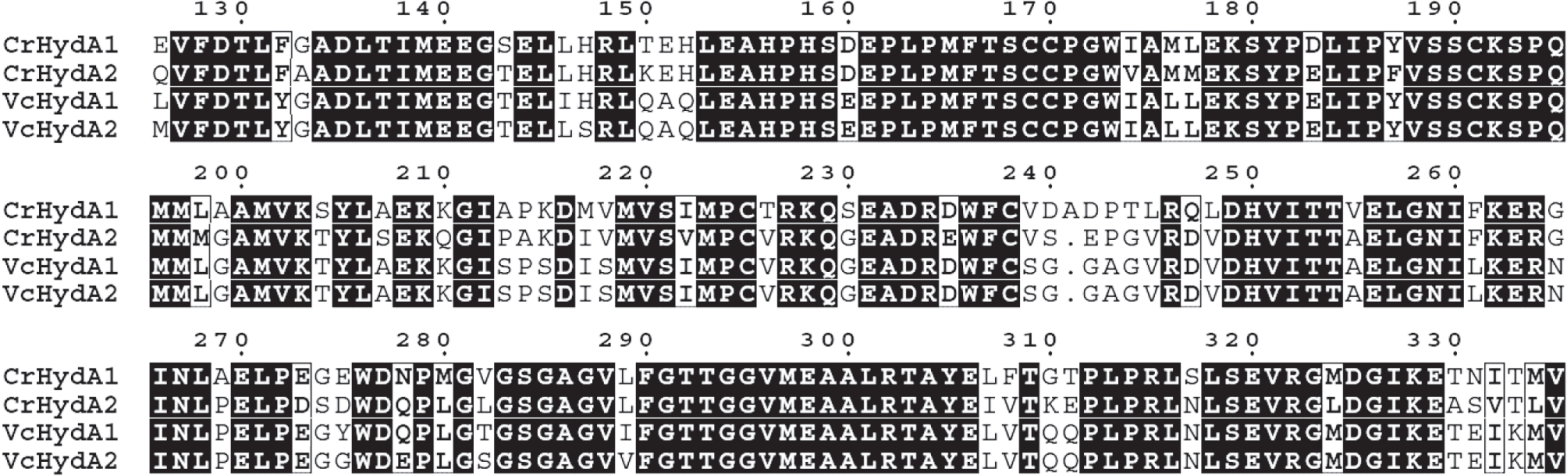
Partial multiple sequence alignment of *V*. *carteri* and *C*. *reinhardtii* HYDA amino acid sequences. Black boxes denote strictly conserved residues, while regions with high conservation are boxed and are in boldface. The HYDA1 proteins from *V*. *carteri* and *C*. *reinhardtii* share 74.1% sequence identity, while the HYDA2 proteins share 75.1% sequence identity. Accession numbers for *C*. *reinhardtii* HYDA1 and HYDA2 are XP_001693376 and XP_001694503, respectively.

*V*. *carteri* is closely related to *C*. *reinhardtii*, a green alga with well-described hydrogen metabolism [28,29]. Recent sequencing and annotation of the *V*. *carteri* genome [31] uncovered two genes with sequence similarity to algal [FeFe]-hydrogenase genes (*HYDA1*, *HYDA2*) (Fig 1) as well as two genes predicted to be necessary for [FeFe]-hydrogenase maturation (*HYDEF*, *HYDG*).

**Fig 2:**
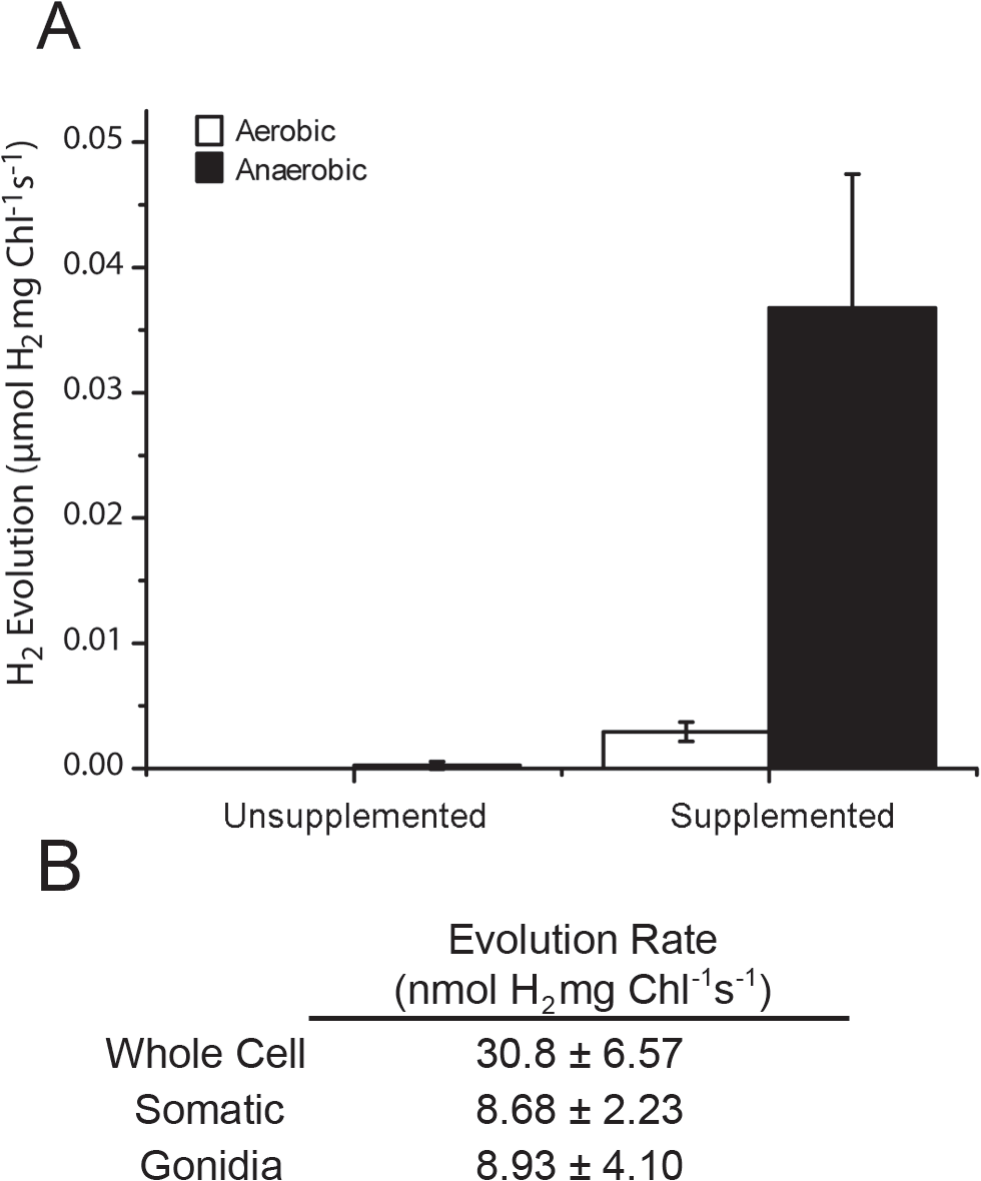
H_2_ evolution of *V*. *carteri* cells. Following a four hour acclimation period, cells were incubated in anaerobic media and H_**2**_ production was measured over a three hour period. The final values were normalized on a per mg chlorophyll basis. A) Whole cell cultures were acclimated to aerobiosis or anaerobiosis and were then supplied either with or without an abiotic electron donor system (Supplemented and Unsupplemented, respectively) during H_**2**_ measurement. Error bars denote standard deviation (n ≥ 3). B) Whole cell cultures and isolated cells (somatic and gonidia) were tested for H_**2**_ evolution activity when provided with an abiotic electron donor system (n ≥ 3).

To assess *V*. *carteri* for *in vivo* and *in vitro* H_2_ production, a culture of cells was acclimated to anaerobiosis for 4 h in degassed media. Aliquots of anaerobically-acclimated cells were transferred to anaerobic assay vials either containing 2 mL of anaerobic media alone or supplemented with an abiotic electron donor (Na_2_S_2_O_4_) and an electron mediator (MV), and the headspace was tested for H_2_ accumulation over time (Fig 2A). H_2_ production could be measured within the first 45 min of incubation with MV/Na_2_S_2_O_4_, eventually achieving an overall integrated production rate of 30.8 nmol H_2_/mg chlorophyll/s after 3 h. Cells that were not supplemented with MV/Na_2_S_2_O_4_ did not accumulate a significant amount of H_2_ in the headspace even 48 h after initiating the assay. When oxygen-exposed cells (i.e. aerobic cells) were assayed under similar conditions, no appreciable H_2_ accumulation was observed over the course of the assay (Fig 2A). These data indicate that, under these conditions, *V*. *carteri* requires both anaerobic acclimation and sufficient reducing equivalents to evolve H_2_.

**Fig 3:**
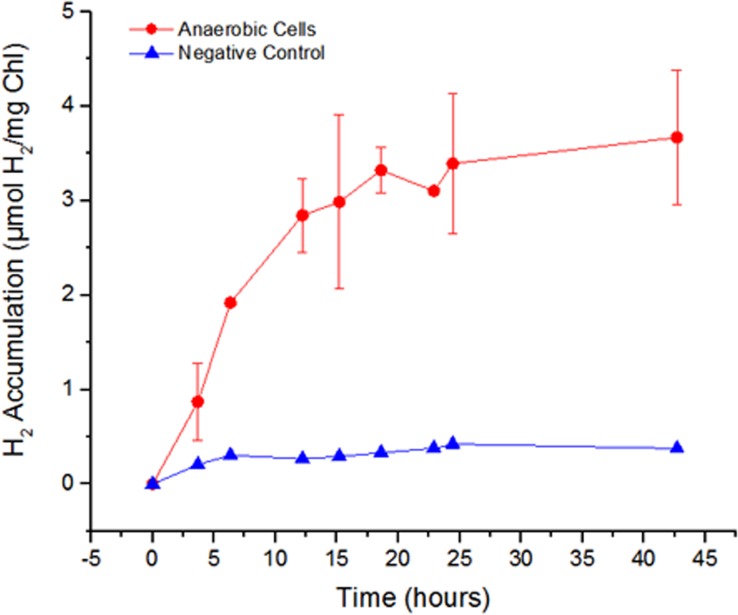
H_2_ production of *V*. *carteri* cells grown with sodium bicarbonate. *V*. *carteri* cultures were incubated with 20 mM sodium bicarbonate-supplemented anaerobic media for 72 hours in the light, then acclimated to anaerbiosis for 4 hours, and assayed for H_**2**_ production under dark anaerobic conditions for 40 hours. H_**2**_ values were normalized to chlorophyll content. (n≥2). The negative control represents unsupplemented cells incubated in anaerobic media which were treated similarly to the cells supplemented with sodium bicarbonate.

In nature, green algae are able to ferment endogenous carbon stores to drive i*n vivo* H_2_ production. However, initially we did not observe appreciable H_2_ accumulation in *V*. *carteri* cells that were anaerobically-acclimated in the absence of Na_2_SO_4_/MV and assayed for H_2_ production. We hypothesized that, under our original growth conditions, fixed carbon levels might be too low to support fermentative H_2_ production. To allow for sufficient carbon fixation to occur, cultures of *V*. *carteri* were supplemented with 20 mM sodium bicarbonate and incubated for 72 hours in the light. The cells were then acclimated to anaerobiosis for four hours and tested for H_2_ formation using unsupplemented SVM as previously described. During the course of the assay, the cells produced appreciable amounts of H_2_ under anaerobic, dark conditions (Fig 3). These results demonstrate that the green alga *V*. *carteri* is competent for H_2_ production under physiological conditions.

### Gonidia and Somatic Cell H_2_ Production

Previous work revealed distinct metabolic differences between the two cell types of *V*. *carteri* [42,47]. To investigate the potential different roles in H_2_ production between the cell types, gonidia and somatic cells were isolated from an anaerobically-acclimated *V*. *carteri* culture and assayed for H_2_ accumulation over a three hour incubation period using the MV/Na_2_S_2_O_4_ electron donor system. H_2_ evolution rates were roughly similar for the two cell types (Fig 2B) nd were ~30% of the rate observed from whole cell extracts on a per chlorophyll basis. The observed difference in the rates may be due to cellular and plastidic damage from the separation procedure. Overall, no major differences in H_2_ evolution rates were observed between the two cell types when supplied with an abiotic electron donor.

### *HYDA* Transcript Analysis

**Fig 4:**
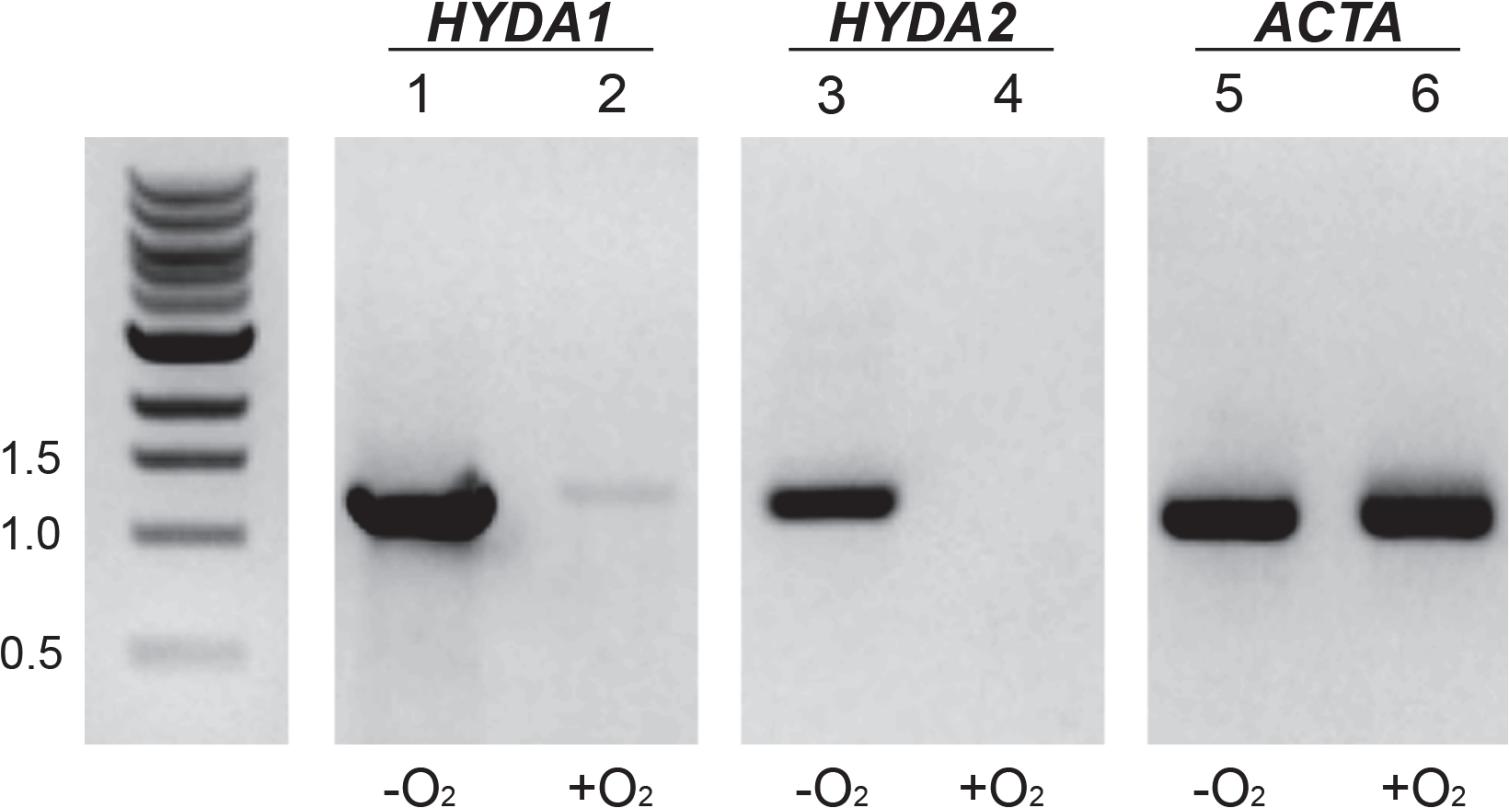
Analysis of *HYDA1* and *HYDA2* genes and gene products. Gene transcript accumulation observed by RT-PCR on a 0.8% agarose TAE gel. Lane 1: *HYDA1* gene amplified from anaerobically-grown *V*. *carteri*. Lane 2: *HYDA1* gene amplified from aerobically-grown *V*. *carteri*. Lane 3: *HYDA2* gene amplified from anaerobically-grown *V*. *carteri*. Lane 4: *HYDA2* gene amplified from aerobically-grown *V*. *carteri*. Lane 5: *ACTA* gene amplified from anaerobically-grown *V*. *carteri*. Lane 6: *ACTA* gene amplified from aerobically-grown *V*. *carteri*. The *ACTA* gene is constitutively expressed under both aerobic and anaerobic conditions and has an amplicon of ~1.1 kb. Molecular weight marker sizes are in kilobases.

We predicted that the putative *HYDA* genes in *V*. *carteri* play a role in H_2_ production. To test for *HYDA* expression during anaerobiosis, RNA was collected from both aerobically- and anaerobically-acclimated cells. The RNA was reverse-transcribed and PCR amplified with primers specific to the putative [FeFe]-hydrogenase genes, *HYDA1* and *HYDA2*. Amplification of mRNA for both genes was observed in anaerobic samples, while very little or no transcript could be observed in the aerobic samples when compared to a housekeeping gene, *ACTA* (accession number XP_002955536) (Fig 4). These results are consistent with the notion that anaerobic expression of *HYDA1* and *HYDA2* correlates with the H_2_ production observed in *V*. *carteri*.

### Hydrogenase Cloning and Characterization

The two putative *HYDA* genes have high sequence similarity to known green algal hydrogenase genes (Fig 1), suggesting that they code for active H_2_-producing enzymes. To test this hypothesis, the two genes were amplified from cDNA, cloned into pAC-BAD, and transformed into *S*. *oneidensis* Δ*hydA*Δ*hyaB* cells. Gene expression was induced by the addition of l-arabinose. The N-terminally His-tagged proteins were purified under strict anaerobiosis by nickel-affinity chromatography and the proteins were analyzed by SDS-PAGE. The HYDA1 and HYDA2 proteins were active when supplied with MV/Na_2_S_2_O_4_, and both had similar *K*_m_ values when measured for MV (2.76 mM and 2.82 mM, respectively). The apparent specific activity for HYDA1 was almost 70-fold greater than for HYDA2 (1,790 nmol H_2_/mg/s and 26.7 nmol H_2_/mg/s, respectively). This differs from previously reported values for recombinant *C*. *reinhardtii* HYDA1 and HYDA2 specific activities, (2,500 nmol H_2_/mg/s and 1,900 nmol H_2_/mg/s, respectively) [48], but is slightly more consistent with the greater than 3.5-fold difference in activity for CpI and CpII of *Clostridium pasteurianum* (2,060 nmol H_2_/mg/s and 7,310 nmol/H_2_/mg/s, respectively) reported by Adams et al. [49].

### Gene Cluster Analysis

**Fig 5:**
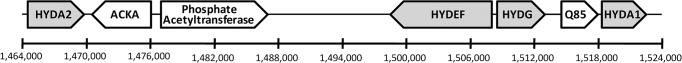
[FeFe]-hydrogenase operon-like gene cluster in *V*. *carteri*. Four genes with sequence similarity to *HYDA1*, *HYDA2*, *HYDEF*, and *HYDG* are arranged within 60 kb of one another. The gene orientation corresponds to the orientation of each shape. Three additional putative genes are within this region: *ACKA* (XP_002948613), a phosphate acetyltransferase (XP_002948484), and a predicted gene, Q85 (XP_002948486). These three predicted gene products are not hypothesized to catalyze H_**2**_ production or assist in the assembly of the [FeFe]-hydrogenase active site, and these genes are not found within investigated bacterial [FeFe]-hydrogenase gene clusters.

The two hydrogenase genes and the two assembly factor genes in the *V*. *carteri* genome (*HYDA1*, *HYDA2*, *HYDEF*, and *HYDG*) were found to be in relatively close proximity to one another. The distance between the furthest genes (*HYDA1* and *HYDA2*) is ~57 kb, and the main cluster of genes is within a ~23 kb span (Fig 5). Although green algae are theorized to have obtained their hydrogenase genes via horizontal gene transfer [24,25,26,27]—likely from an operon or operon-like structure—this level of [FeFe]-hydrogenase gene clustering has not been previously observed in the sequenced genomes of other H_2_-evolving green algae (*C*. *reinhardtii* and *Chlorella variabilis)*.

**Fig 6:**
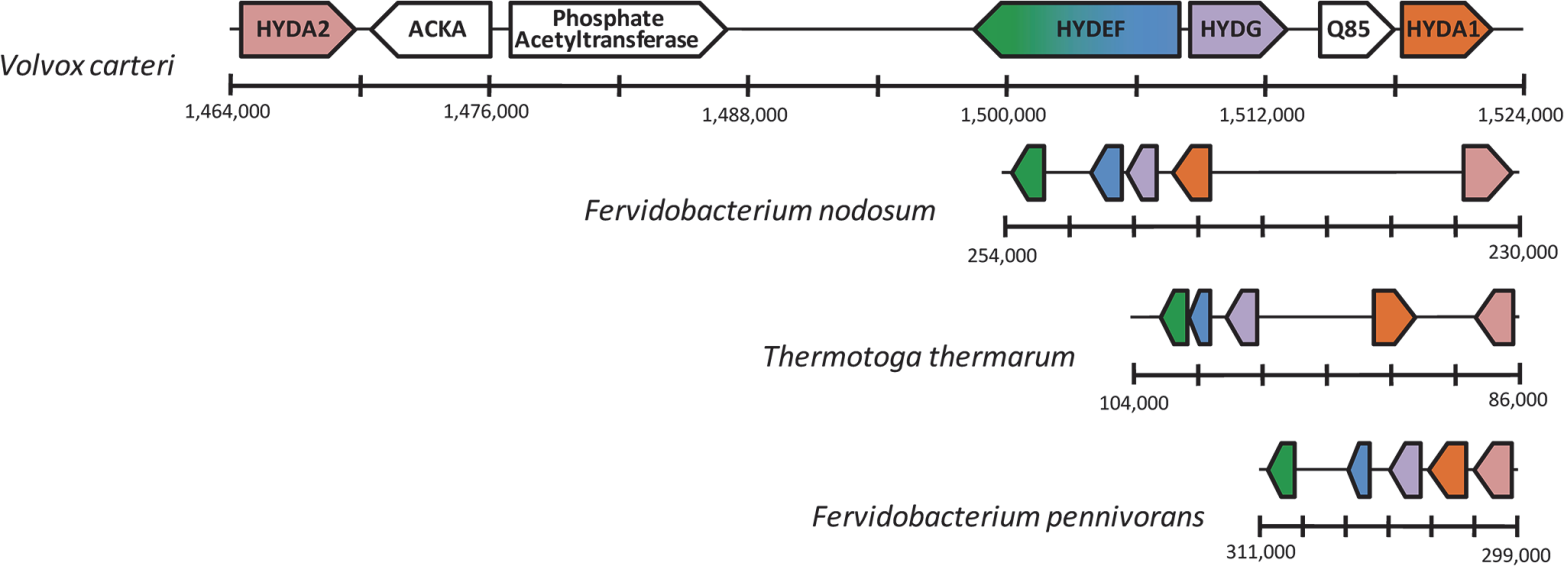
[FeFe]-hydrogenase gene clusters observed in *V*. *carteri*, *F*. *nodosum*, *F*. *pennivorans*, and *T*. *thermarum*. Clusters are all on the same scale, and the putative *HYD* genes are denoted by color (*HYDA* sequences, orange and pink; *HYDE*, green; *HYDF*, blue; *HYDG*, purple; *HYDEF* fusion gene, green-to-blue). The gene orientation corresponds to the orientation of each shape. The coding sequence of the *F*. *nodosum* and *T*. *thermarum* clusters has been reversed (reading 3’ to 5’) to more closely match the orientation of the *V*. *carteri* cluster.

Previously, phylogenetic analyses by Meuser et al. and D’Adamo et al. identified a clade of green algal [FeFe]-hydrogenase genes nested within several bacterial clades [24,25]. Based on these analyses, this clade of hydrogenase genes demonstrated a clear relationship to bacterial genes, strongly indicating an ancestral horizontal gene transfer event. The presence of the hydrogenase gene cluster in *V*. *carteri* could be a remnant of horizontal gene transfer. However, no clustering of [FeFe]-hydrogenase-related genes is observed in the bacterial genomes noted in the clades adjacent to the green algal clade, as observed in the Meuser phylogram [25]. Expanding the search into thermophilic anaerobes, we identified several bacterial genomes which contain gene clusters composed of hydrogenase and hydrogenase maturation genes. In particular, the genomes of *Fervidobacterium nodosum*, *Fervidobacterium pennivorans*, and *Thermotoga thermarum* contain operon-like clusters composed of five hydrogenase genes that cover a <22 kb span (Fig 6, Table 2). It is worth nothing that these clusters demonstrate roughly similar patterns in gene distribution and orientation to each other and the *V*. *carteri* gene cluster.

**Table 2:** Organization and orientation of *HYD* genes in *V*. *carteri*, *F*. *nodosum*, *F*. *pennivorans*, and *T*. *thermarum*.

	Position within Genome					
Organism	*HYDA1*	*HYDA2*	*HYDE*	*HYDF*	*HYDG*	*HYDEF*
*Volvox carteri*	1,518,602–1,522,252 [+]	1,464,508–1,469,778 [+]	na	na	1,508,779–1,513,243 [+]	1,498,998–1,508,353 [–]
*Fervidobacterium nodosum*	244,394–246,154 [+]	231,241–233,444 [–]	248,475–249,524 [+]	252,473–253,684[+]	246,822–248,231 [+]	na
*Fervidobacterium pennivorans*	306,807–308,558 [–]	308,976–310,742 [–]	303,777–304,829 [–]	299,644–300,861 [–]	305,108–306,517 [–]	na
*Thermotoga thermarum*	90,890–92,872 [–]	86,027–87,778 [+]	100,467–101,504 [+]	101,523–102,773 [+]	98,848–100,251 [+]	na

## Discussion

A variety of green algae have been characterized for their ability to couple energy derived from photosynthesis to the production of H_2_, especially the model organism *C*. *reinhardtii* [15,30,50]. Previous work has demonstrated that a species of *Volvox* is capable of H_2_ production [36] to a similar extent as *C*. *reinhardtii*, an alga separated *V*. *carteri* by ~220 million years of evolution. The recently sequenced *V*. *carteri* genome identified two putative [FeFe]-hydrogenase genes [31], prompting us to investigate further the H_2_ metabolism of this organism.

To test the H_2_ metabolism of *V*. *carteri*, algal cell cultures were acclimated to anaerobiosis, while the serum vials were wrapped with aluminum foil, thus eliminating physiological oxygen production and allowing residual oxygen to be removed via respiration. The anaerobic cells were then provided with an electron donor system and H_2_ accumulation was measured. Under the conditions tested, *V*. *carteri* cultures evolved appreciable amounts of H_2_ when supplied with an exogenous electron source (MV/Na_2_S_2_O_4_) to drive H_2_ production.

Previous work has detailed differences in basic metabolism between somatic and gonidia cells, which may have implications related to anaerobic H_2_ production [42,47]. To test for differences in H_2_ metabolism between the two cell types, we separately measured H_2_ evolution activities of isolated gonidia and somatic cells in the presence of an abiotic electron donor. Although the rates of H_2_ evolution in each cell type appeared to be similar (Fig 2B), the H_2_ production rates were normalized based on chlorophyll content. This complicates any direct comparison between gonidia and somatic cells because the amount of photosynthetic machinery does not directly correlate to cell number between the different cell types. Nevertheless, the ability of the isolated cells to generate hydrogen indicates that sufficient hydrogenase machinery is present in both cell types.

Initially, *V*. *carteri* did not demonstrate physiological H_2_ production under the conditions tested. Based on the rationale that cellular carbon sequestration was not sufficient to produce significant quantities of H_2_ during fermentation, algal cultures were supplemented with sodium bicarbonate for 72 hours prior to anaerobic acclimation. In contrast to unsupplemented culture, the sodium-bicarbonate cultures produced demonstrable levels of H_2_, proving that *V*. *carteri* is capable of *in vivo* H_2_ metabolism. The measured H_2_ production reached a maximum of ~3 μmol H_2_/mg Chl at 10 hours, at which point accumulation ceased in the headspace. This is likely due to exhaustion of the fixed carbon storage and represents the maximum yield of H_2_ under these experimental fermentative conditions.

Under anaerobic conditions, transcript for the hydrogenase homologs *HYDA1* and *HYDA2* accumulated in *V*. *carteri*, and these homologs are likely responsible for the measured *in vivo* H_2_ production. In support of this theory, the genes were heterologously-expressed in *S*. *oneidensis* MR-1 and the purified proteins demonstrated quantifiable *in vitro* H_2_ evolution activity when supplied with an artificial electron donor. The purified proteins have similar *K*_m_ values to one another, matching previous literature [48], although the specific activities measured for each enzyme were dissimilar. This discrepancy may be due to incomplete maturation of HYDA2 in the *S*. *oneidensis* MR-1 expression system or may arise from natural differences in activity, as previously observed by Adams et al. [49]. In addition to similar kinetic values, HYDA1 and HYDA2 also share common structural features with other characterized green algal [FeFe]-hydrogenases. For example, both proteins contain two short sequences not observed in non-algal hydrogenases and also lack the canonical iron-sulfur cluster-containing F-domains [14].

While examining the hydrogenase genes within the *V*. *carteri* genome, it was noted that the *HYDA1* and *HYDA2* genes are within close proximity to genes encoding maturation proteins, *HYDEF* and *HYDG* (Fig 5). This level of gene clustering is unique to *V*. *carteri* among the three sequenced green algal genomes, although *HYDEF* and *HYDG* are proximal to each other in the genomes of both *C*. *reinhardtii* and *C*. *variabilis* (S1 Fig). Entire gene clusters can be transferred between organisms during DNA transfer events [51,52,53], and these events can also result in gene fusion, reminiscent of the green algal *HYDEF* fusion [54,55]. [FeFe]-hydrogenase gene clusters, similar to the *V*. *carteri* cluster, were also recently reported in the genomes of the photosynthetic heterokonts *Nannochloropsis oceanica* CCMP1779 [56] and *Nannochloropsis gaditana* CCMP526 [57]. Thus, the acquisition of [FeFe]-hydrogenase genes in heterokonts and green algae may share an evolutionary history, although the exact origin of the *HYD* genes is not known [27,58,59]. Together, the presence of these clusters may provide additional tools to investigate the evolutionary history of [FeFe]-hydrogenases.

Using the phylogenetic analysis established by Meuser et al. [49] as a starting point, we analyzed the genomes of several bacterial species which contained [FeFe]-hydrogenase genes closely related to the *V*. *carteri* genes. Finding that these bacterial genomes did not contain hydrogenase gene clusters, we expanded our search to bacterial genomes not represented in the phylogram by Meuser et al. This search yielded three closely related thermophillic bacterial species (*Fervidobacterium nodosum*, *Fervidobacterium pennivorans*, and *Thermotoga thermarum*) that contained operon-like gene clusters (>22 kb span) within their genomes. It must be noted, however, that distinct differences exist between the *V*. *carteri* and bacterial gene clusters, such as the orientation of the genes and the fusion of the *HYDE* and *HYDF* genes in *V*. *carteri*. It is therefore intriguing that the relative positions of the genes are identical between *V*. *carteri* and bacterial clusters, although the bacterial genes, lacking introns, are shorter in length (Table 2 and Fig 6). Despite these differences, the preserved order of the genes between the *V*. carteri and the bacterial genomes suggests that this clustering may be a remnant of horizontal gene transfer rather than a result of directed gene rearrangements for co-regulation.

Examining the operon-like gene clusters between the thermophiles and *V*. *carteri*—and assuming direct gene transfer from bacteria to green algae—it is tempting to suggest that both *HYDA* genes in green algae were obtained during a single horizontal gene transfer event. In contrast, previous evidence from Meuser et al. would instead indicate that the presence of two *HYDA* genes in *V*. *cateri* result from gene duplication, as these genes are reciprocally monophyletic (Meuser et al. 2011). The presence of the gene cluster in *V*. *carteri*—with both putatively duplicate hydrogenase genes in close proximity to one another—is puzzling, especially as clustering of these genes is not observed in either *C*. *reinhardtii* or *C*. *variabilis*. It is interesting to note, however, that genes encoding phosphate acetyltransferase and acetate kinase are in close association with *HYDA2* in both the *V*. *carteri* and *C*. *reinhardtii* genomes, albeit in different orders in the two species (S1 Fig). This clustering of related genes (acetate assimilation proteins are implicated in fermentative H_2_ production [60]) observed in both species is intriguing, and additional green algal genomes containing hydrogenase genes need to be sequenced to explore the evolutionary history further.

In conclusion, herein we provide the first evidence that *V*. *carteri*, a complex multicellular eukaryote, is capable of producing H_2_ under physiological fermentative conditions. We demonstrate that the two [FeFe]-hydrogenase gene products catalyze H_2_ evolution *in vitro*, suggesting a role for these two enzymes in *V*. *carteri* H_2_ metabolism. A previously unreported gene cluster within the *V*. *carteri* genome encodes these hydrogenases, as well as the essential maturation machinery. This cluster may share a relationship to similar gene clusters found in thermophillic bacteria, providing new avenues in studying the evolutionary origins of [FeFe]-hydrogenases.

## Supporting Information

S1 Fig Representation of *HYD* genes in the *Volvox carteri*, *Chlamydomonas reinhardtii*, and *Chlorella variabilis* sequenced genomes. The gene orientation corresponds to the orientation of each shape. The *HYDEF* and *HYDG* genes are within close proximity with one another in all three genomes, while the *HYDA1* and *HYDA2* genes are only associated with other *HYD* genes in the *V*. *carteri* genome.
(PDF)
