# Supplementary material for: Characterization of Hydrogen Metabolism in the Multicellular Green Alga Volvox carteri
Source: PLoS One. 2015 Apr 30;10(4):e0125324. doi: 10.1371/journal.pone.0125324 (PMC4416025; doi:10.1371/journal.pone.0125324)
Supplement: S1 Fig — The gene orientation corresponds to the orientation of each shape. The HYDEF and HYDG genes are within close proximity with one another in all three genomes, while the HYDA1 and HYDA2 genes are only associated with other HYD genes in the V. carteri genome. (PDF) [file pone.0125324.s001.pdf]

S1 Figure

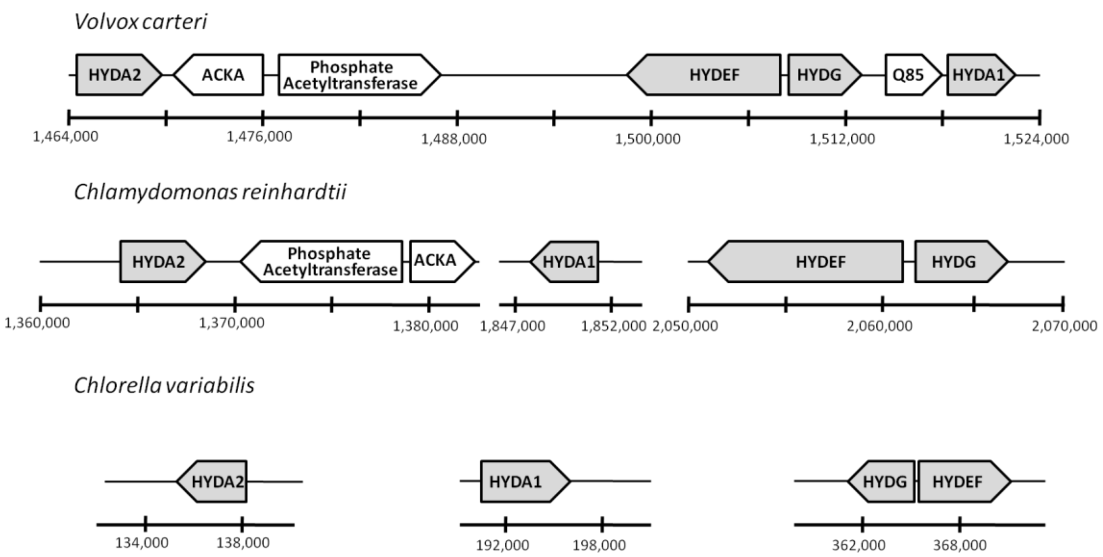

S1 Figure. Representation of *HYD* genes in the *Volvox carteri*, *Chlamydomonas reinhardtii*, and *Chlorella variabilis* sequenced genomes. The gene orientation corresponds to the orientation of each shape. The *HYDEF* and *HYDG* genes are within close proximity with one another in all three genomes, while the *HYDA1* and *HYDA2* genes are only associated with other *HYD* genes in the *V. carteri* genome. Orthologs of acetate kinase (*ACKA*) and phosphate acetyltransferase are in close proximity to *HYDA2* in both the *V. carteri* and *C. reinhardtii* genomes.
